# Placental abruption and kidney disease: evidence for enhanced postpartum surveillance

**DOI:** 10.1016/j.lana.2025.101247

**Published:** 2025-09-12

**Authors:** The Lancet Regional Health – Americas

Acute kidney injury and chronic kidney disease in the postpartum period are overlooked issues in maternal health. One in four pregnant women who have chronic kidney disease suffers a decline in kidney function in the first 6 weeks postpartum. Yet, kidney function is not closely monitored on the postpartum follow-up routine.

Kidney disease is a highly prevalent condition. It is estimated that 9.1% of the global population have chronic kidney disease. In the Americas, the prevalence of kidney disease is also high but varies across regions. For example, the age-standardized prevalence of chronic kidney disease in 2017 was 8144 per 100,000 people in the USA, 7473 per 100,000 people in Andean Latin America, and 11,116 per 100,000 people in Central America. Globally, the age-standardized prevalence of death related to chronic kidney disease in 2017 was 15.9 per 100,000 people.

Around 15% of all pregnant women will develop serious complications, one of which is placental abruption; defined as a premature separation of the placenta during the second half of pregnancy, before delivery. Although placental abruption is rare (occurring in less than 1% of all pregnancies), it is associated with severe bleeding and is the leading cause of maternal and perinatal mortality. One of the maternal complications of placental abruption is acute kidney injury, a sudden loss of kidney function that can cause kidney failure and, in some cases, death. Predisposing risk factors to placental abruption include maternal hypertension and preeclampsia, history of previous placental abruption, maternal trauma, substance use (such as smoking or cocaine), early rupture of membranes, chorioamnionitis, and older maternal age. Perinatal complications include low birthweight, preterm delivery, asphyxia, stillbirth, and death of the foetus. An important gap in the field, however, is whether placental abruption is associated with renal complications (kidney disease and kidney mortality) postpartum.

In our September, 2025, issue, Ananth and colleagues examined the association of placental abruption with kidney disease hospitalization within the first year postpartum. The authors conducted a population-based retrospective cohort study using the Nationwide Readmission Database (nearly 18 million participants) of hospital deliveries and readmissions in the USA, 2010–20 and found that mothers with placental abruption had 70% greater risk of acute kidney injury than did those without abruption. The risk of chronic kidney disease was twice as high in mothers who had abruption than in those without abruption. The risk of acute kidney injury-related mortality among mothers with abruption was four times as high as in those without abruption. The risk of any acute kidney injury or chronic kidney disease (fatal or non-fatal) associated with placental abruption was much greater in those with hypertensive disorders of pregnancy, even after adjustment for multiple covariates, including maternal age, multiple births, preeclampsia, and diabetes. Importantly, mothers with abruption had greater risk of acute kidney injury and chronic kidney disease hospitalizations even in the absence of hypertensive disorders of pregnancy.

The study by Ananth and colleagues suggests the need for close monitoring of kidney function in pregnant women with placental abruption, particularly during the first year postpartum. However, given the retrospective design of their study, with inherent limitations, future randomized controlled trials should be done to confirm these findings and establish causality. Meanwhile, closer postpartum follow-up of mothers with history of placental abruption appears to be justified, at least in the USA. Retrospective and prospective studies should also examine the association between placental abruption and postpartum kidney complications in other populations. If a casual association is confirmed, another aspect that should be considered is whether sufficient resources would be available in other settings (eg, in low-income countries, and rural and underserved areas) to establish effective postpartum follow-up programmes. This is where cost-effectiveness studies will be key, to inform policy makers whether constrained budgets in disadvantaged areas should be allocated to long-term postpartum follow-ups.

Global maternal mortality has declined by 40% since 2020, yet this rate remains very high (197 deaths per 100,000 live births in 2023). Thus, every effort to further reduce the risk of death is worth making, including the prevention of severe kidney injury and fatal kidney disease associated with placental abruption. Unfortunately, little is known about the prevalence of placental abruption in the Americas, offering an opportunity for future research. Ananth and colleagues’ findings might also have a hidden message: we could be missing opportunities to identify potential long-term effects of clinical complications during pregnancy if postpartum follow-ups are not done, a fundamental aspect for improving individual and public health prevention. As we strive for zero maternal deaths, strengthening postpartum surveillance might be a place to start.

